# Electrochemical Biosensor for Rapid Detection of Acute Rejection in Kidney Transplants

**DOI:** 10.1002/adhm.202502831

**Published:** 2025-09-04

**Authors:** Rohit Gupta, Nikolaos Salaris, Ashish Kalkal, Fernando Yuen Chang, Maryam Javed, Azhar Ali Khan, Priya Mandal, Stavroula Balabani, Reza Motallebzadeh, Manish K. Tiwari

**Affiliations:** ^1^ Nanoengineered Systems Laboratory UCL Mechanical Engineering University College London London WC1E 7JE UK; ^2^ UCL Hawkes Institute University College London London W1W 7TS UK; ^3^ Research Department of Surgical Biotechnology Division of Surgery & Interventional Sciences University College London London NW3 2PF UK; ^4^ Department of Nephrology & Renal Transplantation Royal Free Hospital London London NW3 2QG UK; ^5^ FluME UCL Mechanical Engineering University College London London WC1E 7JE UK; ^6^ Institute of Immunity & Transplantation University College London London NW3 2PP United Kingdom; ^7^ Manufacturing Futures Laboratory University College London London E20 2AE UK

**Keywords:** acute rejection, antifouling, electrochemical biosensor, kidney disease monitoring, point‐of‐care diagnostics

## Abstract

Kidney transplant recipients face a high risk of acute rejection (AR), where the immune system attacks the transplanted organ. Current diagnostics rely on invasive biopsies with procedural risks, costs, and limited temporal resolution. While urinary chemokines CXCL9 and CXCL10 are promising non‐invasive AR biomarkers, clinical adoption is limited by labor‐intensive detection and lack of point‐of‐care (POC) solutions. A rapid, label‐free electrochemical biosensing platform for simultaneous quantification of CXCL9 and CXCL10 chemokines from 5 µL of unprocessed urine in 15 min, which for ELISA and biopsy is between 24–72 hrs, is presented. The system uses screen‐printed carbon electrodes modified with a Ti_3_C_2_T_x_ MXene‐crosslinked bovine serum albumin hydrogel, offering high conductivity, nano‐porosity, anti‐fouling properties, and signal stability for up to 30 days. The platform enables single‐digit pg/mL‐level sensitivity, meeting clinical thresholds. In a prospective clinical study, biosensor‐measured chemokine data trained a bootstrapped logistic regression classifier, achieving 83% AR classification accuracy. When combined with additional clinical and histopathological features, accuracy increased to 98%. This work integrates advanced materials, biosensor engineering, and machine learning to deliver a scalable, cost‐effective POC solution for real‐time, non‐invasive AR monitoring. The platform will help reduce biopsy dependence, enable earlier intervention, and ultimately improve long‐term transplant outcomes.

## Graphical abstract

A low‐cost, rapid electrochemical immunosensor coated with a novel antifouling nanocomposite enables single‐step, dual‐biomarker profiling directly from unprocessed urine. Application in a clinical study shows accurate discrimination of acute rejection in kidney transplants from other acute kidney injuries via machine learning. This offers a potential alternative to invasive biopsies and complex multi‐step assays.

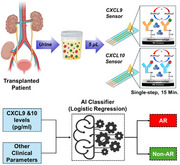

## 1 Introduction

Kidney transplantation is a critical, life‐saving intervention for patients with end‐stage kidney disease (ESKD). Despite advances in surgical techniques and immunosuppressive therapies, long‐term graft (transplant) survival remains a significant challenge, with up to 20% of recipients experiencing immune‐mediated injuries.^[^ ^1^, ^2^, ^3^ ^]^ Acute rejection (AR) manifests as a rapid inflammatory response driven by T‐cells and/or donor‐specific antibodies (DSAs) targeting cellular allo‐antigens and graft endothelial cells.^[^ ^3^ ^]^ Although AR rarely leads to immediate graft loss,^[^ ^4^, ^5^ ^]^ it can represent an independent risk factor for chronic rejection and shortened long‐term graft survival,^[^ ^6^ ^]^ as well as for premature death, particularly from cardiovascular disease and cancer.^[^ ^7^, ^8^ ^]^ There are currently no effective therapies for chronic rejection, suggesting that the key to improving long‐term graft prognosis is to both detect early and achieve remission of early AR events to prevent activation of irreversible chronic inflammatory pathways.

Currently, percutaneous needle biopsy remains the gold standard for clinical management and accurate diagnosis of AR.^[^ ^9^, ^10^ ^]^ However, biopsies are invasive, carry risks such as bleeding, and are subject to sampling errors and interpretative variability. These limitations, coupled with the logistical challenges of frequent monitoring, underscore the urgent need for non‐invasive diagnostic alternatives. In practice, serum creatinine (sCr), proteinuria levels, and the presence of DSA are often used to direct biopsies for confirmation of AR.^[^ ^11^, ^12^, ^13^ ^]^ However, these lack the required sensitivity and specificity, which can lead to delayed intervention, with substantial graft injury occurring sub‐clinically (before sCR elevation).^[^ ^14^ ^]^ Urinary biomarkers that can predict AR early and are easy to measure would improve clinical decision making and help either avoid a biopsy, or direct one at an earlier time point compared to that governed purely by the current measures.^[^ ^14^, ^15^ ^]^

Recent advances in proteomics have identified urinary C‐X‐C motif chemokines CXCL9 and CXCL10 as promising biomarkers for AR,^[^ ^16^, ^17^ ^]^ capable of distinguishing between T‐cell‐mediated^[^ ^18^ ^]^ and antibody‐mediated rejection subtypes,^[^ ^19^ ^]^ and critically, at a stage when graft injury is reversible. They outperform standard post‐transplant surveillance by detecting early AR before elevation of sCr.^[^ ^15^ ^]^ Integrating urinary CXCL9 and CXCL10 levels into clinical workflows has been shown to reduce the need for biopsies by as much as 60%.^[^ ^20^ ^]^ Although enzyme‐linked immunosorbent assays (ELISA) have been utilized to measure these biomarkers, they require multi‐step protocols and batched processing, and exhibit limitations such as interference from complex biological matrices. Even though several other works have consistently detected urinary CXCL9 and CXCL10 in patients using commercially available ELISA kits, the horseradish peroxidase (HRP) enzyme precipitations interfere with several inhibitory compounds in urine (e.g., urea, peroxide substances, glutathione, albumin, etc.) increasing the chances for erroneous measurements.^[^ ^21^ ^]^ These challenges make ELISA unsuitable for point‐of‐care (POC) applications for rapid and routine AR monitoring, and individual assessment of the efficacy of anti‐rejection therapies.

Electrochemical biosensors (EBs) offer a transformative solution for rapid, sensitive, and multiplexed biomarker detection.^[^ ^22^ ^]^ By integrating antibody‐functionalized electrodes with advanced nanomaterials (e.g., gold nanoparticles,^[^ ^23^ ^]^ ZnO,^[^ ^24^, ^25^ ^]^ or magnetic nanomaterials^[^ ^26^ ^]^), EBs can achieve high detection sensitivity in the picogram range. However, existing EB platforms often face signal interference from non‐specific interactions in complex biological samples such as unprocessed urine, limiting their performance and applicability.^[^ ^27^ ^]^ Recent developments in anti‐biofouling electrode coatings with tuneable electrical conductivities offer promising solutions to these challenges.^[^ ^28^ ^]^ 3D nanoporous hydrogels using crosslinked bovine serum albumin (BSA) combined with electroconductive materials (gold nanowires,^[^ ^28^ ^]^ carbon nanotubes, or reduced graphene oxides^[^ ^29^, ^30^, ^31^ ^]^) have shown excellent anti‐biofouling properties for EB platforms. Nonetheless, EBs developed with these nanomaterials almost always require additional amplification steps using enzyme‐tagged detection antibodies. This increases turnaround time, assay complexity, and associated costs.^[^ ^28^, ^31^ ^]^

Last year, a Ti_3_C_2_T_x_ MXene‐filled BSA hydrogel nanocomposite reported the feasibility of chemokine detection in spiked serum albumin samples, showcasing anti‐biofouling properties and cost‐reduction potential.^[^ ^32^ ^]^ However, clinically relevant point‐of‐care (POC) diagnostics of AR require rapid detection in unprocessed urine, containing a diverse range of proteins (100 Da to 50 kDa) that may cause non‐specific binding and false positives. Additionally, integrating sensor outputs with automated classification is crucial for early clinical intervention in AR cases.

Here, we combine an MXene/BSA hydrogel‐based EB platform for rapid dual‐chemokine profiling coupled with a probabilistic logistic regression classifier, showcasing the possibility of diagnosing acute AR rejection directly using patient urine and in 15 minutes. Thus, the platform enables non‐invasive chemokine‐based diagnostics over conventional ELISA and biopsies with long turnaround times (24–72 hrs). This system retains anti‐biofouling properties, enhances areal capacitance, requires no additional amplification, and achieves a single‐digit pg/mL analyte detection. In a clinical study of 60 kidney transplant recipients, benchmarking against ELISA and biopsy‐confirmed outcomes demonstrated that our sensor, combined with the binary artificial intelligence (AI) classifier, distinguishes AR from other kidney transplant pathology with up to 98% accuracy. Each recipient underwent a biopsy to determine the cause of acute graft dysfucntion—including antibody‐mediated AR, T‐cell‐mediated AR, mixed AR, or non‐acute rejection—highlighting the platform's clinical use potential.

## 2 Results

### 2.1 Electrochemical Behaviour of Nanocomposite‐modified Electrode

**Figure 1:**
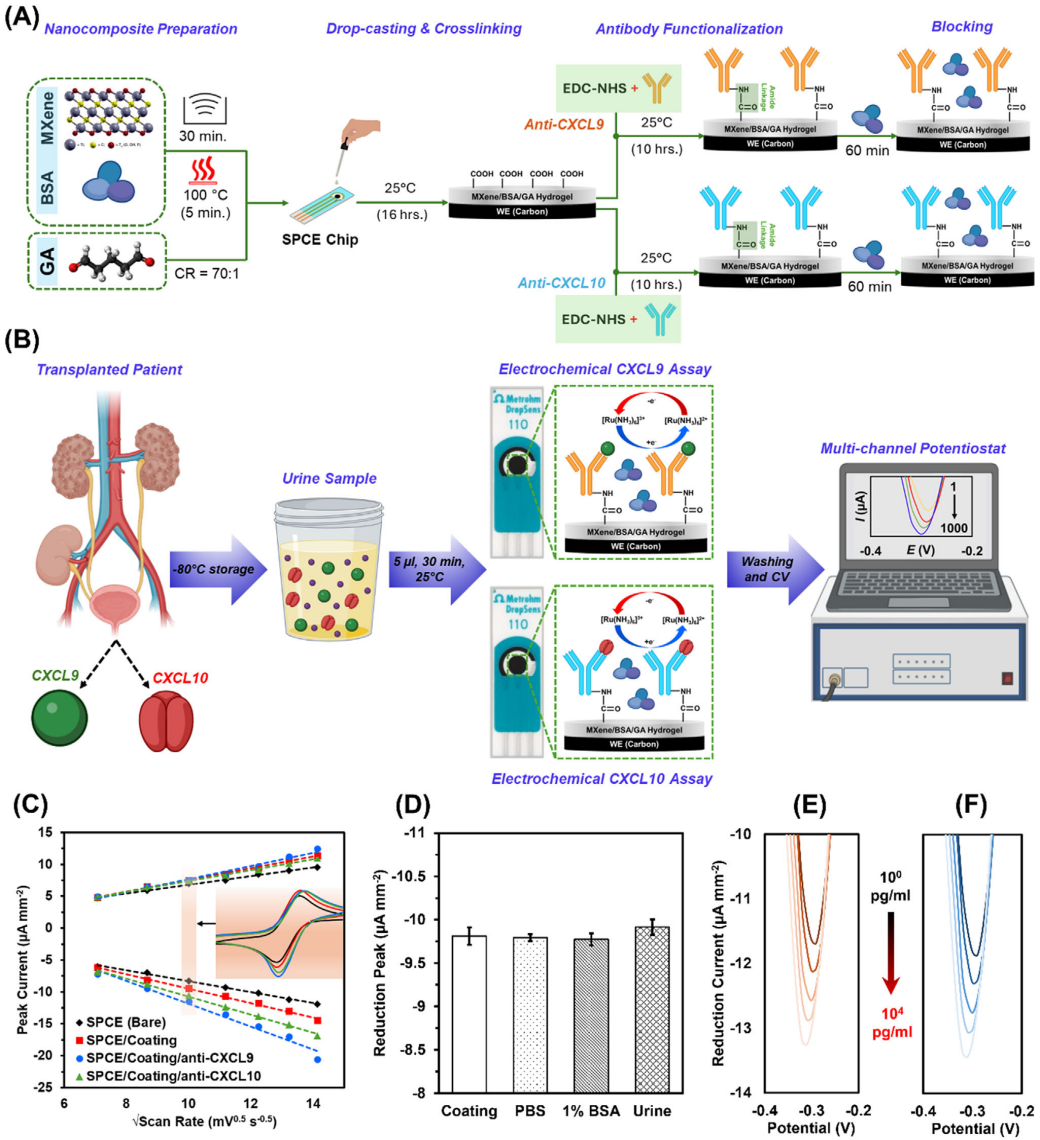
Development and characterization of the electrochemical sensor. A) Fabrication of the CXCL9 and CXCL10 assays using SPCE modified with MXene/BSA/GA nanocomposite. B) Detection protocol starting from urine collection to signal extraction. C) Scan rate dependent peak current responses of bare, nanocomposite (coating)‐modified, anti‐CXCL9 functionalized, and anti‐CXCL10 functionalized SPCEs (inset shows CV responses at 100 mV s^−1^). D) Reduction peak currents of MXene/BSA/GA‐coated SPCEs when incubated for 24 h with PBS, 1% BSA, and undiluted urine sample (healthy non‐transplanted donor). Error bars represent ±1 SD of mean of *n* = 3 independent electrodes. Statistical significance analysis revealed non‐significant difference across all the samples, indicating no electrode passivation. E,F) Reduction current responses obtained from the label‐free CXCL9 and CXCL10 sensing assay, respectively, upon incubating different concentrations (1–10^4^ pg mL^−1^) target chemokines spiked in healthy non‐transplanted urine sample. Both the assays show monotonically increasing reduction peak current as a function of target chemokine concentration.

Nanointerfaces composed of Glutaraldehyde (GA)‐crosslinked BSA hydrogels with conductive nanofillers (e.g., AuNW, CNT, rGO) are known to exhibit strong antibiofouling properties while enabling high redox diffusion.^[^ ^28^, ^29^, ^30^ ^]^ To further enhance the faradaic and capacitive performance of these interfaces, Ti_3_C_2_T_x_ MXene was used as a substitute nanofiller, resulting in the development of the novel MXene/BSA/GA composite. This nanocomposite was applied to the working electrode (WE) of a commercial screen‐printed carbon electrode (SPCE) chip and analyzed using cyclic voltammetry (CV) with [Ru(NH_3_)_6_]^3^⁺ as the redox probe (see Figure 1). The linear relationship between the faradaic peak current density and the square root of the scan rate observed across a broad scan rate range (50–200 mV s^−1^) in **Figure** **1**C confirms that the MXene/BSA/GA interface supports diffusion of the electroactive [Ru(NH_3_)_6_]^3+/2+^ species seamlessly.^[^ ^37^ ^]^ The increase in peak‐to‐peak separation (ΔE_pp_) from 100 to 120 mV after nanocomposite coating suggests quasi‐ (i.e., partially) reversible electron transfer. Importantly, subsequent functionalization with specific antibodies, such as anti‐CXCL9 or anti‐CXCL10, did not inhibit faradaic current peaks, indicating that the nanointerface maintains porous diffusion characteristics, suitable for label‐free single‐step biosensing applications.

To evaluate the antibiofouling properties of the MXene/BSA/GA, the uncoated carbon WE and MXene/BSA/GA‐coated carbon WE were compared following their exposure to different concentrations of albumin for a period of 24 h (Figure S2, Supporting Information). In the case of uncoated electrodes, progressive increases in albumin concentration (0 to 100 mg mL^−1^) resulted in up to a 35% decrease in Faradaic peak current, indicating significant electrode passivation due to nonspecific protein adsorption. In contrast, the MXene/BSA/GA‐coated electrodes maintained stable Faradaic signals across the same concentration range, suggesting effective resistance to nonspecific adsorption. Furthermore, the coated electrodes were exposed to a drop of undiluted urine sample collected from a healthy non‐transplanted donor, for which the Faradaic peak current changed less than 2% (Figure 1D), reconfirming the biomolecule adsorption resistance of the MXene/BSA/GA coating.

Subsequently, MXene/BSA/GA‐coated WEs functionalized with anti‐CXCL10 and anti‐CXCL9 were tested with variable CXCL10 and CXCL9 protein concentrations (1–10⁴ pg mL^−1^), respectively, spiked in urine from healthy donors. Each chip was incubated with ≈10 µL of the spiked urine for up to 15 min at room temperature, followed by washing phosphate buffer saline (PBS) and CV analysis using [Ru(NH_3_)_6_]^3^⁺ at a scan rate of 100 mV s^−1^ (Figure 1E). Increasing CXCL9 concentrations consistently correlated with a rise in reduction peak current near −0.3 V, indicating reduced charge transfer resistance and improved electron transfer as the antigen‐antibody complex forms. This phenomenon is attributed to the anti‐CXCL9 functionalized MXene/BSA/GA nanocomposite, which initially acts as a pre‐charged capacitor, impeding electron transfer from the redox probe. Upon CXCL9 binding, surface charge redistribution occurs, partially neutralizing the capacitive charge, reducing impedance, and thus enhancing the observed peak current.^[^ ^38^ ^]^ Similar sensing characteristics were observed for the CXCL10 sensor over for protein concentrations between 1–10⁴ pg mL^−1^, indicating identical sensing mechanism (Figure 1F).

### 2.2 Understanding the Morphology, Surface Topography, and Cross‐linking

**Figure 2:**
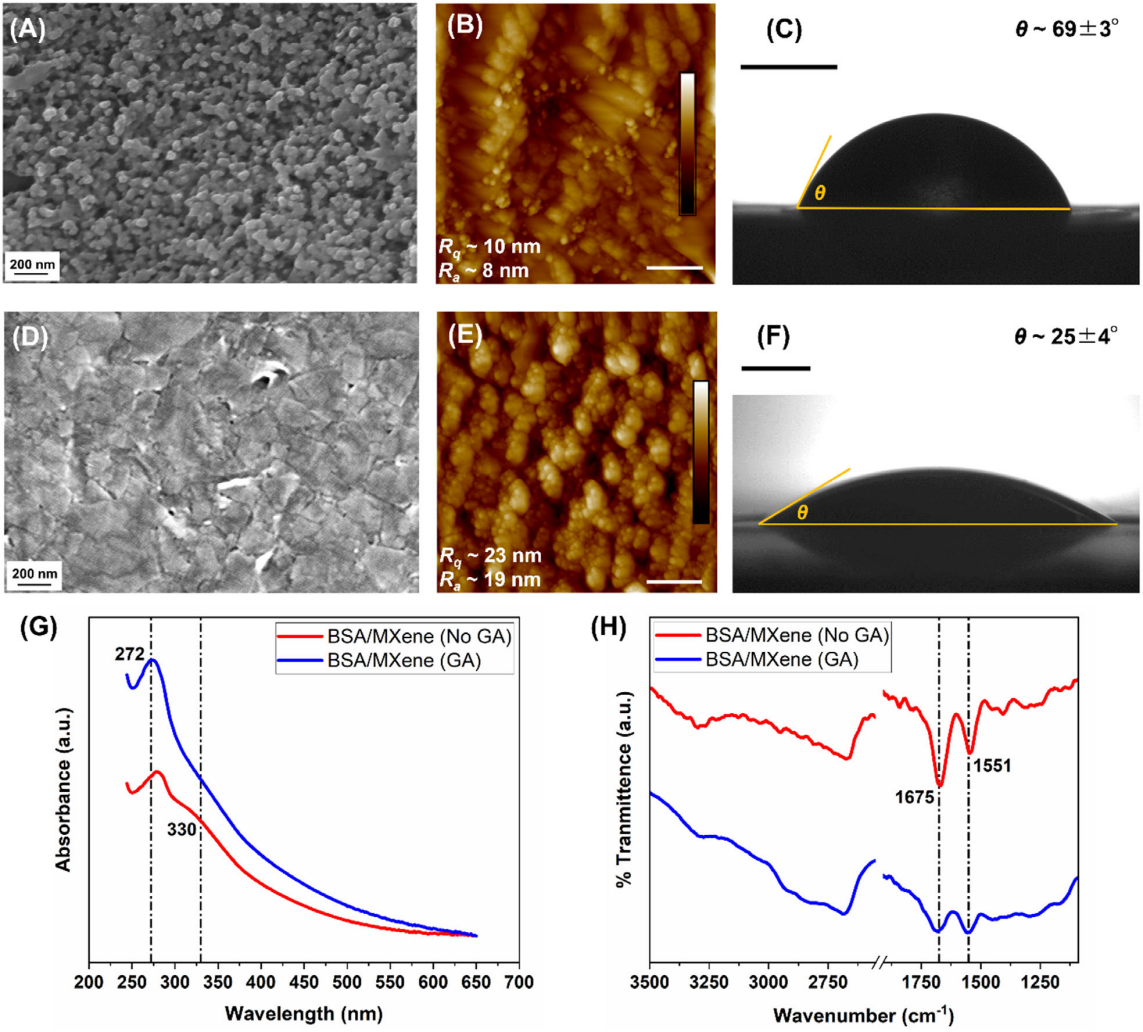
Morphological and chemical characterization of the MXene/BSA/GA‐based biosensor interface. A) SEM, B) AFM, and C) static CA of bare carbon WE. D) SEM, E) AFM, and F) static CA of nanocomposite‐modified WE. G) UV‐Visual spectroscopy showing the confirmation of MXene/BSA crosslinked through GA evidenced via peak amplification at 272 nm and shoulder disappearance at 330 nm wavelengths. H) FTIR spectroscopy revealing the amide bond (amide I and II) formation upon GA‐mediated crosslinking of the hydrogel. Scale bars in (B) and (E) measure 500 nm, while those in (C) and (F) measure 1 mm.

To characterize the surface morphology of the bare WE of SPCE chips and those coated with MXene/BSA/GA nanocomposite, scanning electron microsopy (SEM) was employed. This revealed a densely packed BSA/GA matrix embedded with Ti_3_C_2_T_x_ MXene nanoflakes (**Figure** **2**A,D). Additionally, atomic force microscopy (AFM) was used to assess the surface roughness and porosity of the nanocomposite layer. The root mean squared roughness (*R_q_*) and average roughness (*R_a_*) values, measured over a 2 × 2 µm^2^ area, were 10 and 8 nm, respectively, for the bare WE, compared to 23 and 18 nm for the MXene/BSA/GA‐coated chips (Figure 2B,E). The cross‐linked nanocomposite features nanometer‐sized pores that improve the electrode's electrochemical performance by forming isolated mesopores, which function as nanoelectrodes and boost faradaic peak currents.^[^ ^28^ ^]^ Additionally, the fine porosity (<10 nm) of the MXene/BSA/GA coating serves as a size‐exclusion filter, reducing interference from non‐specific proteins in complex biological samples, which could otherwise passivate the electrode surface.

Beyond surface morphology, the wetting characteristics of the nanocomposite provide insights into its antibiofouling properties. Ideal antibiofouling surfaces exhibit reduced adsorption of non‐specific proteins, which can impede the diffusion of electroactive species by passivating the electrode surface.^[^ ^39^ ^]^ Hydrophilic surfaces reduce non‐specific protein adsorption by forming an interfacial hydration layer, which limits the likelihood of non‐covalent interactions with biomolecules.^[^ ^28^ ^]^ As shown in Figure 2C,F, the MXene/BSA/GA coating reduced the contact angle (CA) by 44° compared to the bare WE, indicating a significant enhancement in surface hydrophilicity. This pronounced hydrophilic behavior, combined with the negatively charged functional groups inherent to Ti_3_C_2_T_x_ MXene,^[^ ^39^ ^]^ contributes to the observed antifouling performance through a combination of hydration‐mediated shielding and electrostatic repulsion. Together, these properties support stable Faradaic responses, even in biological samples like unprocessed urine.

The GA‐mediated cross‐linking of the MXene/BSA nanocomposite was analyzed by Ultraviolet‐visual (UV‐Vis) and Fourier‐transform Infrared (FTIR) spectroscopies (Figure 2G,H). BSA/GA crosslinking occurs via solvent‐accessible lysine residues, forming imine bonds that establish a porous 3D nanointerface.^[^ ^28^ ^]^ This cross‐linking reaction is evident through amplification of within the wavelength band of 265–275 nm. In addition, the absorbance shoulder at 330 nm wavelength for the native MXene/BSA disappears after GA cross‐linking. Both indicates structural changes in the peptide backbone and the formation of a homogenous conjugated protein network. FTIR analysis further provides chemical characteristics of the GA cross‐linking, mechanism where BSA/MXene samples without GA exhibit distinct amide‐I (1675 cm^−1^) and amide‐II (1551 cm^−1^) stretches due to C = O stretching and N‐H bending/C‐N stretching, respectively. Upon successful GA crosslinking, the intensity of these vibrations is diminished, indicating consumption of the functional groups while cross‐linking.

### 2.3 Analytical Performance of Dual Biomarker Sensing Platform

Using MXene/BSA/GA‐coated SPCE chips, two types of electrochemical sensors were created to detect CXCL9 and CXCL10 protein concentrations in urine samples (see Figure 1B). Each sensor was functionalized with antibodies specific to either CXCL9 or CXCL10. The sensors were then exposed to urine samples from healthy controls (non‐transplanted donors) or to samples spiked with known concentrations of the respective proteins. This exposure lasted for 15 min at room temperature to allow for antigen‐antibody binding. After this incubation, the sensors were washed with PBS, and CV measurements were performed at a scan rate of 100 mV s^−1^. For each measurement, a 100 µl droplet containing 10 mM [Ru(NH_3_)_6_]^3^⁺ and 100 mM KCl in PBS was applied, and the reduction peak current density, normalized to the electrode geometric area of 12.56 mm^2^, was recorded at −0.3 V as the electrochemical signal.

**Figure 3:**
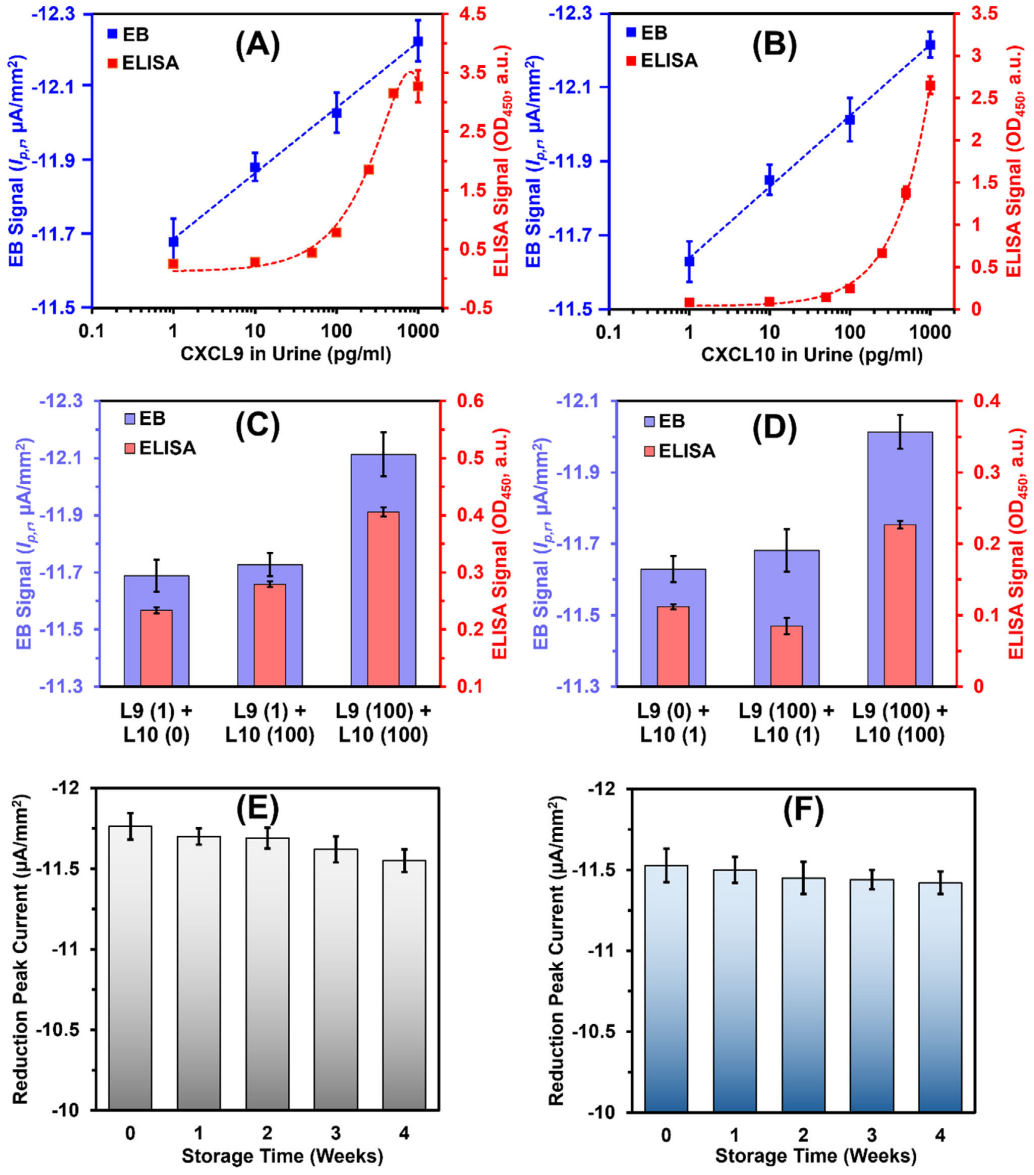
Key performance metrics of the electrochemical sensor. Calibration curves for EB (reduction peak current, µA/mm^2^) and ELISA (absorbance at 450 nm) assays for A) CXCL9 and B) CXCL10 proteins, spiked in urine samples from healthy donor. The negative current value signifies reduction peak. Intraassay cross‐reactivity tests for EB and ELISA‐based C) CXCL9 and D) CXCL10 assays, tested via simultaneously spiked 0, 1 or 100 pg mL^−1^ of CXCL9 or CXCL10 proteins. The numbers inside the brackets indicate the amount of different protein present (e.g., 0, 1, or 100 pg mL^−1^) in different samples. E,F) Stability test CXCL9 and CXCL10 sensors, respectively, stored at 4 °C and investigated periodically over 1‐month. Error bars in all the graphs represent ±1 SD of mean for *n* = 3 independent electrochemical chips or ELISA wells.

The results demonstrated that the reduction peak current densities for both CXCL9 and CXCL10 sensors showed a logarithmically linear response as antigen concentrations rose (**Figure** **3**A,B). For comparison, parallel measurements conducted using ELISA exhibited a linear response to increasing antigen concentrations. The log‐linear response for EBs suggest that they have higher sensitivity at lower analytes ranges (i.e., 1–100 pg mL^−1^). This enhanced sensitivity arises from: a) the electroactive nanocomposite amplifying current signals, b) antibiofouling properties minimizing non‐specific adsorption, and c) direct assay format enabling target binding near the electrode. In contrast, ELISAs lack dedicated antibiofouling coatings and rely on sandwich formats with reduced dual‐binding efficiency at low analyte concentrations, limiting sensitivity in the low pg/mL^−1^ range. The electrochemical sensors achieved limits of detection (LOD) for CXCL9 and CXCL10 of 6.9 and 5.7 pg mL^−1^, respectively, while the sandwich ELISA assays showed comparable LODs of 3.9 and 4.5 pg mL^−1^. In healthy individuals, typical concentrations of CXCL9 and CXCL10 range between 5 to 25 pg mL^−1^, whereas in patients with acute kidney disease, these levels can rise by an order of magnitude.^[^ ^16^, ^17^ ^]^ Consequently, the electrochemical sensor's log‐linear detection range of 7–1000 pg mL^−1^ effectively spans the clinically relevant range for these biomarkers.

The MXene/BSA/GA coating demonstrated significant efficacy in mitigating biofouling‐induced signal passivation, thereby improving sensor specificity. However, further validation of cross‐reactivity is critical to ensure minimal interference between the antigen‐antibody interactions of CXCL9 and CXCL10. To address this, cross‐reactivity between these biomarkers was evaluated using both electrochemical and ELISA‐based methodologies (Figure 3C,D). Anti‐CXCL9 functionalized chips exposed to urine samples containing 100 pg mL^−1^ of CXCL10 exhibited a negligible peak current density increase of 0.4% (Figure 3C), signifying low cross‐reactivity. In contrast, samples containing both CXCL9 and CXCL10 at 100 pg mL^−1^ showed a peak current density increase of 3.3% (Figure 3C), confirming the specific binding of CXCL9 antigens.

Based on the abovementioned cross‐reactivity tests, the electrochemical assays for CXCL9 and CXCL10 achieved signal‐to‐noise ratios (SNR) of 9.1 and 6.7, respectively, indicating strong specific responses compared to non‐specific responses. Comparatively, the ELISA‐based assays exhibited significantly higher non‐specific responses (19.5% for CXCL9 and 24% for CXCL10), resulting in reduced SNRs of 3.8 for CXCL9 (*p* < 0.001) and 4.3 for CXCL10 (*p* < 0.001).

Furthermore, for each of the CXCL9 and CXCL10 sensing chips, controlled experiments were performed using a wide range of biomolecules and electrolytes pertinent to urine samples (e.g., albumin, ascorbic acid, creatinine, uric acid, glucose, sodium, potassium, calcium) that may interfere with the EB signal (Figure S3, Supporting Information). The maximum interference in the peak currents from these non‐specific compounds were found to be only 2.7% and 2.5% for CXCL9 and CXCL10 sensing signals, respectively. These findings highlight the superior performance of the present label‐free electrochemical sensing approach enhanced by the electroactive antibiofouling nanocomposite. This protocol effectively improved sensor SNR, highlighting its potential for precise and reliable biomarker detection.

The shelf‐life of the antibody‐functionalized SPCE chips was evaluated by storing them at 4 °C for up to four weeks and periodically monitoring their performance, as illustrated in Figure 3E,F. Over this period, both sensors maintained a stable electrochemical response for up to three weeks, with a maximum signal degradation of less than 5%, indicating strong performance stability over time.

### 2.4 Rapid Urinary CXCL9 and CXCL10 Profiling in Clinical Samples

**Figure 4:**
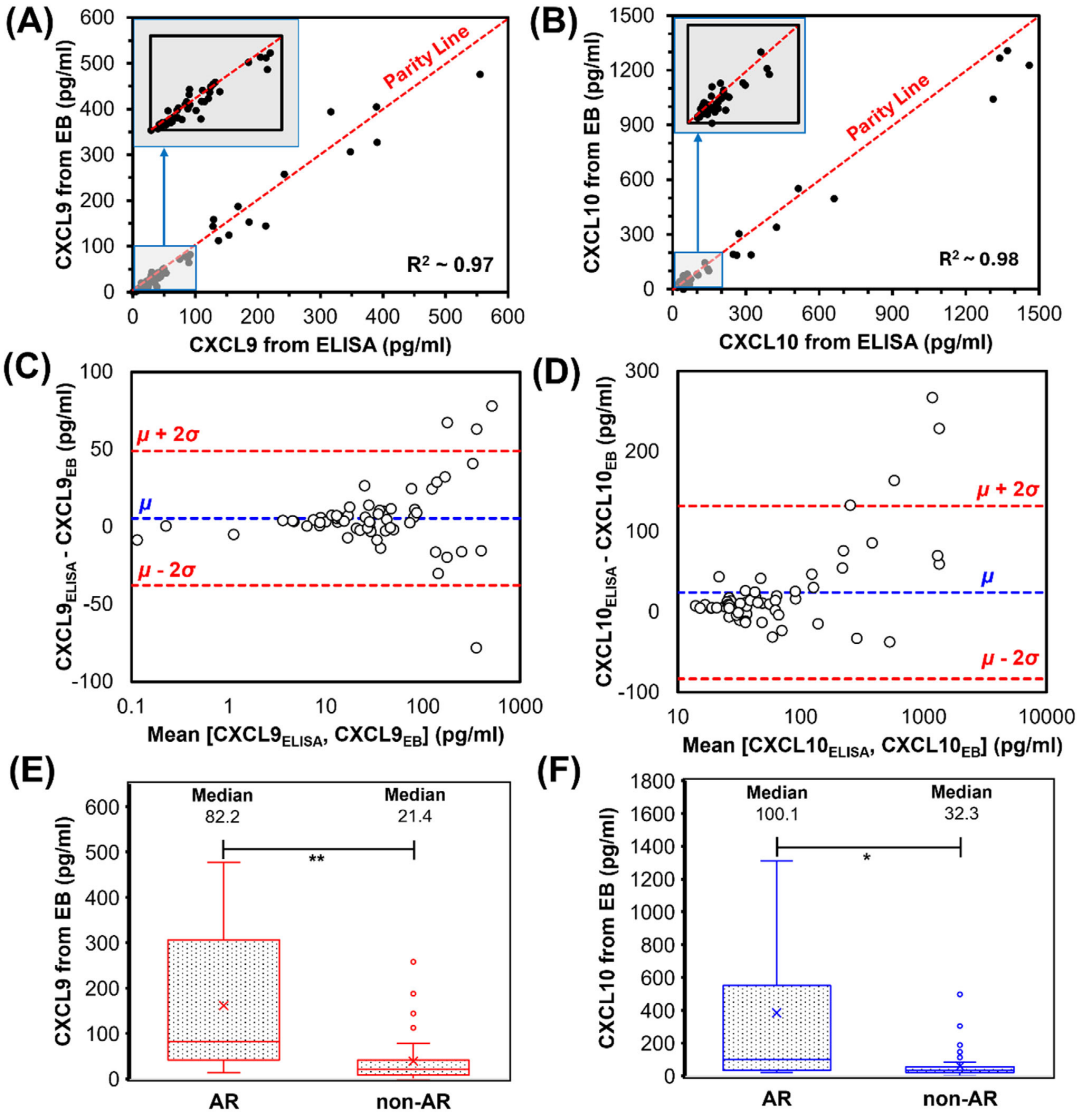
Performance of the electrochemical sensor in clinical study with *n* = 60 kidney transplanted patients being diagnosed for AR. Gold‐standard method (ELISA) against the EB for measuring A) CXCL9 and B) CXCL10 in urine sample for the patient cohort. Bland‐Altman plots further describing the agreement between ELISA and EB methods for C) CXCL9 and D) CXCL10, respectively. Mean (µ) line represents the bias of EB sensor µ ± 2σ are the limits of agreement with 95% confidence interval. Box‐Whisker plots showing E) CXCL9 and F) CXCL10 levels measured by EB for AR and non‐AR groups in within the patient cohorts. Circles outside the box represent the outliers, lines within the box denote 25th, 50th (median), and 75th percentile, respectively, and cross marker is the mean for each group. Median values for each chemokine clearly distinguish between AR and non‐AE groups. Significant difference was determined by unpaired two‐tailed *t*‐test assuming unequal variances (*p<0.05, **p<0.01).

Having established the performance of our novel sensor in spiked urine, a double‐blind study profiling CXCL9 and CXCL10 was conducted on unprocessed urine samples from 60 transplant recipients with and without biopsy‐proven AR, utilizing both EB and ELISA‐based methods. Details of the electrochemical assay protocol are presented in Section 3.3, while the ELISA was conducted with minor modifications to the manufacturer's instructions (see Section 2.5). The measured CXCL9 and CXCL10 concentrations ranged from 0 to 600 pg mL^−1^ and 0 to 1300 pg mL^−1^, respectively, as illustrated in the scatter plots (**Figure** **4**A,B). Linear regression analysis demonstrated strong agreement between the electrochemical sensor and ELISA measurements, yielding adjusted R^2^ values of 0.97 for CXCL9 and 0.98 for CXCL10, respectively. The agreement between the two methods was particularly robust at concentrations below 100 pg mL^−1^, as confirmed by Bland‐Altman plots (Figure 4C,D). The mean bias (µ) for the CXCL9 sensor was 5.4 pg mL^−1^, with 95% limits of agreement (µ ± 2σ) at 48.8 and −37.9 pg mL^−1^, where σ represents the standard deviation. Similarly, for the CXCL10 sensor, µ, µ + 2σ, and µ – 2σ values were 24.2, 131.8, and −83.5 pg mL^−1^, respectively. High sensitivity at low biomarker concentrations (<200 pg mL^−1^) is advantageous for the early detection of AR. Notably, for the CXCL9 and CXCL10 EBs, four and three samples, respectively, fell outside the ±2σ limits of the ELISA benchmark in the Bland‐Altman analysis, indicating some deviation in chemokine quantification. Upon closer inspection, these outliers correspond to samples with chemokine concentrations exceeding 200 pg mL^−1^. In this range, the sensor response transitions from linear to logarithmic, potentially reducing sensitivity and contributing to the observed deviations.

Biopsy‐confirmed diagnoses were used to categorize patient samples into AR and non‐AR groups. The distribution of CXCL9 and CXCL10 concentrations in these groups is shown in Figure 4E,F. A clear distinction in measured concentrations of CXCL9 (*p* < 0.01) and (*p* < 0.05) CXCL10 biomarkers between non‐AR and AR groups show the potential of these biomarkers to differentiate between the two groups with high confidence. However, a small subset of outliers (5 out 60 patients, i.e., 8% of the dataset) was observed in the non‐AR group, suggesting the need for further clinical scrutiny as they may represent other renal pathologies.

In four of the five outlier cases, biopsy results indicated acute tubular injury, most associated with delayed graft function (DGF), a complication frequently observed in the early period following kidney transplantation. DGF is primarily driven by ischemia‐reperfusion injury, which results from prolonged cold ischemic times (i.e., the duration during which the donor kidney is preserved at low temperatures prior to transplantation). This process can cause damage to the tubular epithelium and trigger inflammation, thereby inducing the release of local pro‐inflammatory chemokines such as CXCL9 and CXCL10. These findings align with previous clinical studies^[^ ^40^, ^41^ ^]^ and likely explain a subset of chemokine‐positive, non‐AR cases within our dataset. In future studies, incorporating time‐dependent chemokine profiling post‐transplantation may help to distinguish these cases more accurately.

A separate confounding factor, identified in one of the five outlier cases, was BK polyomavirus (BKPyV) infection. BKPyV is a well‐recognized cause of elevated urinary CXCL9 and CXCL10 levels, in the absence of AR.^[^ ^15^, ^42^ ^]^ As such, active BKPyV infection represents a potential diagnostic blind spot when relying solely on urinary chemokine measurements. However, this form of infection can be independently verified through plasma polymerase chain reaction (PCR) testing, making it clinically distinguishable from immune‐mediated allograft rejection.

### 2.5 Data‐Driven AR Classification

**Figure 5:**
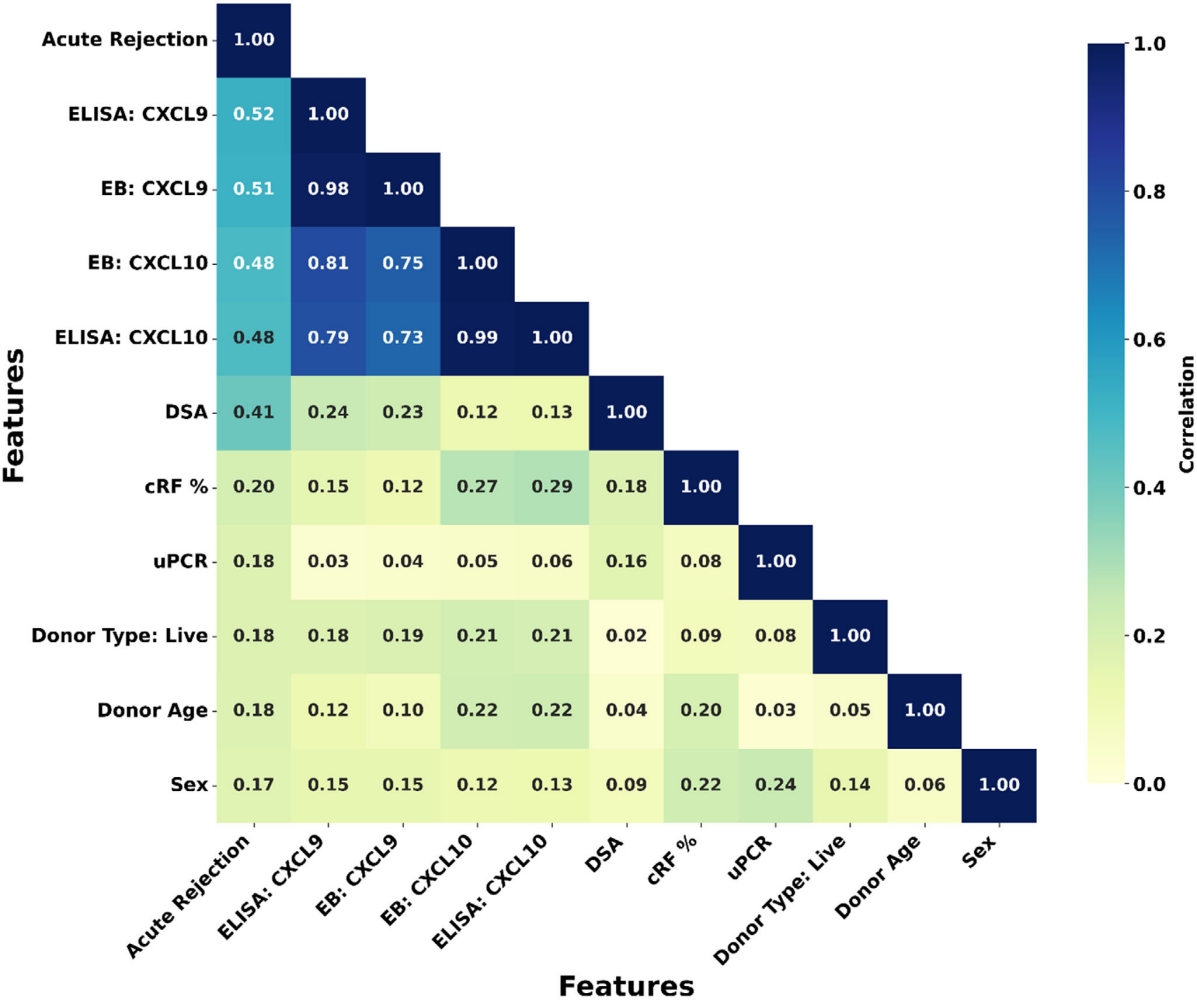
Heatmap showing pairwise Pearson Correlation Coefficient (PCC) between the 10 most important predictive features and biopsy outcome. Both the horizontal (x‐axis) and vertical (y‐axis) axes list the model's predictors. The color of each square corresponds to the absolute correlation value, as indicated by the color bar on the right, where darker colors signify a stronger linear relationship (PCC ∼ 1) and lighter colors indicate a weaker relationship (PCC ∼ 0). This is a measure of the strength of the linear relationship between each pair of features.

Exploratory analysis of the dataset, visualized via a pairwise Pearson Correlation Coefficient (PCC) heatmap (**Figure** **5**), revealed strong correlations between urinary biomarker levels (CXCL9 and CXCL10, measured via both EB and ELISA) and biopsy outcomes (PCC > 0.5). This indicates their potential as robust biomarkers for AR classification. Furthermore, a strong intercorrelation between the two chemokines were observed (0.7 < PCC < 0.8), suggesting their complementary usability.^[^ ^43^ ^]^ Among clinical variables, presence of DSA and level of allo‐sesitization (i.e., calculated reaction frequency, cRF%) demonstrated a notable correlation with biopsy outcomes with PCC values of 0.41 and 0.2, respectively. Other factors such as urinary protein‐to‐creatinine ratio (uPCR), donor type, donor age, and sex exhibited weaker correlations with PCC < 0.2.

**Figure 6:**
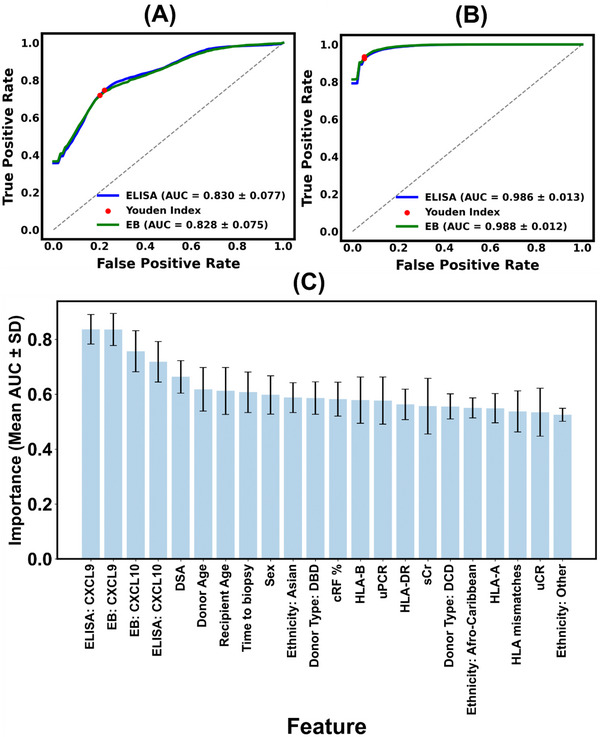
Receiver Operating Characteristic (ROC) curves, evaluating the diagnostic accuracy of classification model. A) Performance of classifier that utilizes only CXCL9 and CXCL10 levels as predictors to predict biopsy outcome. B) Classifier model performance built using chemokine levels and other parameters from the clinical database (total 13 continuous and 4 categorical predictors) to predict biopsy outcome. The red dots indicate the optimal classification threshold determined by the Youden Index. In both A,B), the dashed line represents the performance of a random classifier. C) Histogram ranking the predictors by their decreasing importance. The y‐axis shows the mean AUC‐ROC value for each individual feature, calculated across 1000 bootstrap iterations. Error bars represent the ±1 SD of the mean of *n* = 1000 AUC values from the bootstrap.

The predictive utility of CXCL9 and CXCL10 as urinary biomarkers for was further assessed using logistic regression models. ROC curve analysis demonstrated comparable classification performance for both ELISA and assays. For the EB‐based chemokine model, the area under curve (AUC) of receiver operating characteristic (ROC) curve was 0.83 (±9.05%), while the corresponding ELISA‐based model achieved a similar AUC of 0.83 (±9.3%). It was furthermore confirmed that chemokine‐based classification model did not overfit since AUC_bootstrap_ ≈ AUC_validation_ (Figure S4, Supporting Information). Using the Youden Index, the true positive rate (TPR) and false positive rate (FPR) were determined to be 0.72 and 0.20, respectively, for the EB, and 0.75 and 0.22 for the ELISA assay (**Figure** **6**A). Adding subsequent features to the model progressively enhanced the model performance. For example, the model with the five most significant predictor variables achieved an AUC value of 0.901 (±5%) with TPR and FPR of 0.83 and 0.17. Similarly, the model with the 10 most significant features achieved an AUC of 0.94 (±3.1%), yielding TPR and FPR of 0.87 and 0.14 (Figure S4, Supporting Information). Nevertheless, both the models with the five and ten features showed significant overfitting as the AUC_bootstrap_ > AUC_validation_, which would significantly warrant expanding the patient population size in future work.

Incorporating all 17 predictor variables into a comprehensive model substantially improved predictive accuracy but resulted to overfitting (AUC_bootstrap_ > AUC_validation_). This multivariable model yielded AUC values of 0.987 (±1.3%) for both EB‐ and ELISA‐based measurements. The FPR was reduced to 0.05 for both methods, while the TPR increased to 0.93 for EB and 0.92 for ELISA (Figure 6B). The sensitivity, specificity, positive predictive value (PPV), and negative predictive value (NPV) for the comprehensive model were 0.972, 0.962, 0.916, and 0.988, respectively.

Feature importance analysis based on AUC calculations confirmed that CXCL9 and CXCL10 were the most influential predictors of biopsy outcomes (Figure 6C). Clinical factors such as presence of DSAs, recipient and donor ages, time to biopsy, and donor type were alsoimportant parameters, emphasizing their relevance in AR prediction. Other factors, including cRF%, ethnicity, sex, level of human leukocyte antigen (HLA) mismatch, and uPCR provided further incremental improvements.

These findings highlight the utility of combining biomarker assays with clinical variables to develop a comprehensive and clinically relevant diagnostic tool for AR prediction. Validation through larger patient cohorts is recommended to confirm these results and further refine the model's diagnostic capabilities.

## 3 Discussions and Concluding Remarks

The current standard for diagnosing acute rejection (AR) in kidney transplant recipients, namely biopsies and ELISA‐based assays, presents significant limitations in clinical practice. Biopsies are invasive, expensive (£1000–£3000), and can delay diagnosis by several days, while ELISA assays, though less invasive, yet expensive (£50‐£150 per ELISA) require laboratory infrastructure, trained personnel, batch processing and prolonged processing times (24–72 h).^[^ ^44^ ^]^ These delays may result in missed therapeutic windows and increase the risk of irreversible graft injury.

In the UK alone, diagnostic spending related to kidney transplant rejection, including an estimated 1050 annual biopsies (i.e., 30% of new transplant recipients/year) exceeds £2.4 million.^[^ ^45^ ^]^ A rapid, point‐of‐care diagnostic tool could significantly reduce this burden. Even considering 50% adoption could save over £1.2 million annually, not including downstream costs from delayed or unnecessary interventions. Broader deployment across transplant types and healthcare systems underscores the potential for substantial economic and clinical impact.

To address these challenges, we developed a rapid electrochemical biosensing platform capable of detecting CXCL9 and CXCL10 directly from unprocessed urine, costing <£1.2 per chip. These chemokines are associated with T‐cell and antibody‐mediated rejection, respectively, offering diagnostic and prognostic value. The platform features a single‐step assay that eliminates the need for detection antibodies, enzymes, or chromogenic substrates. Integration of Ti_3_C_2_T_x_ MXene within a BSA/glutaraldehyde hydrogel enhances conductivity and biofouling resistance, enabling robust performance. Clinical validation showed strong concordance with ELISA (R^2^ > 0.95).

Further, we implemented a binary classification model using dual‐chemokine data (i.e., CXCL9 and CXCL10) to differentiate AR from other acute kidney injuries. Diagnostic accuracy reached 83%, increasing to 98% with the inclusion of clinical features such as DSA, level of HLA mismatch, and donor type; exceeding the performance of conventional markers like eGFR and proteinuria (AUC‐ROC 0.54–0.72).^[^ ^16^, ^17^, ^18^, ^46^ ^]^

Despite these promising results, limitations include a relatively small cohort (*n* = 60), underscoring the need for multi‐center trials to evaluate generalizability for AR monitoring and sub‐cohort analysis (e.g., distinguishing between antibody‐mediated, T‐cell mediated and mixed rejections). Additionally, progress from manual sample handling to automated fluidics and transitioning to antibody‐free sensors is essential for clinical scalability. Further work is also required for integrating automated sample processing, a flow‐cell interface, on‐board reagents, molecularly imprinted polymer to replace antibodies, cloud‐connected potentiostat, and advanced ML algorithms to enable point‐of‐care deployment.

In conclusion, this study presents a significant advance in the non‐invasive, real‐time diagnosis of AR in kidney transplant recipients. By combining rapid, accurate chemokine detection with machine learning and user‐friendly design, this platform lays the foundation for personalized, proactive transplant care. Its integration into clinical practice holds the promise of reducing invasive diagnostics, improving patient outcomes, and redefining the standard of care in kidney transplantation.

## 4 Experimental Section

### Reagents and Materials

Delaminated Ti_3_C_2_T_x_ MXene nanoflakes were obtained from Nanoplexus Ltd. Screen‐printed carbon electrode (SPCE) chips (DropSens 110), featuring a three‐electrode system (working electrode (WE): carbon, counter electrode (CE): carbon, and reference electrode (RE): silver chloride), were purchased from Metrohm Ltd (see Figure 1A,B). Hexaammineruthenium(III) chloride and potassium chloride were sourced from Chemlab Ltd. Phosphate‐buffered saline (PBS) tablets, human anti‐CXCL10 antibody (CHC2363), human CXCL10 protein (CHC2363), 2‐(N‐morpholino)ethanesulfonic acid (MES), and N‐hydroxysuccinimide (NHS) were supplied by Thermo Fisher. Human CXCL9 protein and anti‐CXCL9 antibody (900‐K87K) were procured from PeproTech. Bovine serum albumin (BSA), 70% glutaraldehyde (GA), N‐ethyl‐N′‐(3‐dimethylaminopropyl)carbodiimide (EDC), and ethanolamine were obtained from Sigma Aldrich. All reagents were prepared in 10 mM PBS (pH≈7.2) using DI water unless otherwise specified.

### Nanocomposite Preparation

Ti_3_C_2_T_x_ MXene nanoflakes were dispersed in DI water through probe sonication for 2 h to produce a 4 mg mL^−1^ suspension. Simultaneously, a 50 mg mL^−1^ (5 wt.%) BSA solution was prepared in PBS. A mixture containing 5 mg mL^−1^ BSA and 0.5 mg mL^−1^ MXene was then bath sonicated for 30 min with 1 s on/off pulses, followed by heating at 100 °C for 5 min to denature the BSA (as depicted in Figure 1A). The mixture was centrifuged at 1000 rpm for 15 min to remove aggregates, after which 1% GA was added at a cross‐linking ratio of 70:1 (i.e., 69 µl of MXene/BSA mixed with 1 µl of 70% GA) to form the cross‐linked MXene/BSA/GA nanocomposite.^[^ ^28^ ^]^

### Dual‐Chemokine Electrochemical Sensor Fabrication

The fabrication process of SPCE‐based nanocomposite‐coated CXCL9 and CXCL10 EB is shown in Figure 1A. Approximately 5 µl of the MXene/BSA/GA composite was drop‐cast onto the WE and incubated overnight in a humidified chamber at room temperature to form a 3D nanostructured hydrogel coating. The electrodes were then washed with PBS under agitation (400 rpm) to remove excess materials and dried with a gentle N_2_ stream. Anti‐CXCL9 and anti‐CXCL10 antibodies were immobilized on the SPCE chips using carbodiimide crosslinking chemistry. Specifically, a solution containing 400 mM EDC, 200 mM NHS, and 50 µg mL^−1^ of either anti‐CXCL9 or anti‐CXCL10 antibody was prepared in 50 mM MES buffer (pH≈6.2) and agitated on an orbital shaker at room temperature for 90 min to activate the antibody functional groups.^[^ ^33^ ^]^ A 5 µl aliquot of the activated antibody solution was then applied to the nanocomposite modified WE and incubated overnight in a humidified chamber. Following antibody immobilization, unreacted GA in the nanocomposite was quenched using 1 M ethanolamine for 30 min, followed by a 1 h incubation with 1 wt.% BSA. Each fabrication step included washing with ≈3 ml PBS and drying with N_2_. The antibody‐functionalized electrodes were stored at 4 °C until further use.

### Morphological, Chemical, and Electrochemical Characterizations

The morphology of the nanocomposite coated WE of SPCEs were investigated using scanning electron microscopy (SEM) and atomic force microscopy (AFM). For SEM analysis, a thin (<5 nm) gold coating was applied to the composite via sputtering. SEM imaging was conducted with a Zeiss EVOLS15 instrument, utilizing an in‐lens detector to collect secondary electrons under an accelerating voltage of 10 kV. AFM imaging was performed on a Bruker Multimode 8‐HR in non‐contact tapping mode, using tips with an approximate curvature radius of 20 nm. Surface roughness was quantified through average (*R_a_*) and root mean‐squared roughness (*R_RMS_*) values, determined using the NanoScope 2.0 software.

The crosslinking characteristics of the MXene/BSA/GA nanocomposite were evaluated using UV–vis and Fourier transform infrared (FTIR) spectroscopies. For UV–vis measurements, MXene/BSA solutions, both pre‐ and post‐GA addition, were diluted 25‐fold to minimize excessive UV absorption at the lower spectral range.^[^ ^28^ ^]^ FTIR characterization of the crosslinked MXene/BSA/GA was conducted with a Nicolet iS50 FTIR spectrometer. Transmittance spectra were obtained from powdered samples over the wavenumber range of 600–3000 cm^−1^, with each spectrum averaged over 32 scans.

Cyclic voltammetry (CV) was performed using an IviumStat electrochemical workstation within a potential window of −0.6 to 0.1 V, with a scan rate of 100 mV s^−1^ and a potential step of 10 mV. The electrochemical response was measured with a 100 µl droplet of a solution containing 10 mM [Ru(NH_3_)_6_]^3^⁺ and 100 mM KCl in PBS, and the average voltammogram from three cycles was reported. The current response, recorded in µA was converted into current density (µA/mm^2^) based on the geometric area of the WE, which was 12.56 mm^2^.

### ELISA‐based CXCL9 and CXCL10 Measurements

The sandwich ELISA assays for CXCL9 and CXCL10 detection in urine samples were conducted using NUNC MaxiSorp flat‐bottom transparent 96‐well plates, following the manufacturer's instructions for the CXCL10 (CHC2363, Invitrogen) and CXCL9 (900‐K87K, PeproTech) ELISA kits. Anti‐human CXCL9 and CXCL10 capture antibody stocks were diluted to 1 and 2 µg mL^−1^, respectively, in 1X coating buffer (prepared from 10X PBS, Invitrogen). A volume of 100 µL of the diluted antibody was added to each well, after which the plate was sealed with parafilm to prevent evaporation and incubated overnight at 4 °C. Following incubation, the wells were washed three times with 1X wash buffer (prepared from 20X wash buffer with 0.05% Tween 20 in 1X PBS) to remove unbound capture antibody. To block non‐specific binding sites, 200 µL of 1X assay buffer (prepared from 5X ELISA/ELISPOT buffer, Invitrogen) was added to each well, followed by a 2 h incubation at room temperature. After blocking, the wells were washed three times with 1X wash buffer. Urine samples spiked with CXCL9 and CXCL10 from a non‐kidney transplanted healthy donor was added to separate wells as control samples (100 µL per well) for the calibration curve, while urine samples from kidney transplant patient cohort were added to the remaining wells. After a 2 h incubation at room temperature, wells were washed three times with 1X wash buffer. Next, 100 µL of biotin‐conjugated anti‐human CXCL9 and CXCL10 detection antibodies, diluted to 1 and 0.2 µg mL^−1^, respectively, in 1X assay buffer, were added to each well, followed by a 1 h incubation at room temperature. Wells were washed four times, and a streptavidin‐conjugated HRP solution (diluted to 1/2000 and 1/2500, specific to CXCL9 and CXCL10 ELISA kits, respectively, in 1X assay buffer) was added at 100 µL per well. Following a 30 min incubation at room temperature, wells were washed five times. The reaction was developed by adding 100 µL of TMB substrate (Invitrogen) to each well and incubating in the dark for 15 min. The enzymatic reaction was then stopped by adding 100 µL of 1 M H_2_SO_4_ stop solution to each well, stabilizing the yellow endpoint. Absorbance was measured at 450 nm using a Tecan Infinite 200 Pro microplate reader.

### Study Population and Clinical Samples

This study received ethical approval from the National Research Ethics Services (NRES) Research Ethics Committee (REC, approval number 21/WA/0388) and the Royal Free Biobank (ethical reference NC.2018.010). Urine samples (≈2 mL) were collected from pseudo‐anonymized kidney transplant patients (*n* = 60) at the Royal Free Hospital, London, undergoing for cause of biopsies (at the discretion of the patient's physician) and who gave a written informed consent. Immunosuppression included induction with anti‐CD25 monoclonal antibody and maintenance immunosuppression with a calcineurin inhibitor, and mycophenolic acid with or without corticosteroids. Urine samples obtained just before biopsies were performed, frozen at −80 °C immediately after collection and stored for up to 36 months before analysis. Bacterial (e.g., urinary) and BK virus infections were routinely tested for in patients with acute renal allograft dysfunction as per standard of care. Both ELISA and EB platforms used undiluted, unprocessed clinical urine samples. A comprehensive clinical dataset including patient demographics, clinical laboratory test results, biopsy indications, transplant variables, and outcomes was constructed for the recruited patients (Table S1, Supporting Information). The diagnosis of AR was established using the Banff 2019 criteria, with all biopsies examined by a renal histopathologist.

### Statistical Analysis

All EB and ELISA signals are reported as mean of replicates (*n* = 3), with error bars representing the standard deviation (SD) of the mean. The standard calibration curves in the urine matrix were fitted with a linear function for ELISA and a logarithmic function for EB. Agreement between ELISA and EB was evaluated using coefficient of determination (R^2^) and Bland‐Altman plots with 95% limits of agreement (i.e., µ ± 2σ). Statistical significance was assessed using unpaired, two‐tailed t‐tests assuming unequal variances, with significances levels defined as p < 0.05 (*) and p < 0.01 (**). All analyses were conducted in Origin2023 software.

For the continuous clinical variables other than CXCL9 and CXCL10 levels (see Table S1, Supporting Information), descriptive statistics were reported as mean (± SD) or median with interquartile range (IQR), as appropriate. Categorical variables are reported as percentages of the total clinical dataset (*n* = 60). Pearson Correlation Coefficient (PCC) were used to quantify pairwise linear correlations between variables across dataset.^[^ ^34^ ^]^

### Classification Model Development

A classification model was developed to differentiate AR from other forms of kidney transplant pathologies. This model incorporated CXCL9 and CXCL10 levels (measured via EB or ELISA), along with the following continuous clinical variables: serum creatinine (sCR, µmol/L), urinary creatinine (mg/dL), urinary protein‐to‐creatinine ratio, human leukocyte antigen (HLA) mismatch level, calculated reaction frequency (cRF; %), transplant‐to‐biopsy interval (years), recipient age (years), and donor age (years). Categorical variables included: presence of donor‐specific antibodies (DSA; Yes/No), bacteriuria exceeding 10⁴ CFU/mL (Yes/No), donor type (living/circulatory‐death/brain‐death), recipient ethnicity (White/Asian/Afro‐Caribbean/Other), and recipient sex (male/female). The model outcome variable was binary (AR versus non‐AR), as determined by histopathological diagnosis. Detailed descriptions of the clinical dataset were provided in Table S1 (Supporting Information).

For preprocessing, missing values in continuous variables were imputed using the mean, and missing categorical values were imputed using the mode. One‐hot encoding was applied to categorical variables to enable their inclusion in the regression‐based classification model.^[^ ^35^ ^]^ Given the limited sample size (*n* = 60), outlier removal was not performed.

Model performance was assessed using the area under the receiver operating characteristic curve (AUC‐ROC) to quantify discrimination between AR and non‐AR. Optimal cut‐off points were determined using the Youden Index,^[^ ^36^ ^]^ and additional performance metrics included sensitivity, specificity, positive predictive value (PPV), and negative predictive value (NPV).

Feature importance was estimated via a bootstrap‐based logistic regression approach to reduce overfitting. In each of 1000 iterations, the dataset was resampled with replacement to generate a bootstrap cohort. A logistic regression model was trained on each cohort, and AUC‐ROC scores were computed from the predictions. This produced a distribution of AUC‐ROC values for each predictor, from which mean and standard deviation were recorded. Further details for the complete modelling workflow along with the source code was provided in Figure S1 (Supporting Information). The model was implemented in Python 3.9 using *pandas*, *scikit‐learn*, *statsmodels*, *matplotlib*, and *seaborn*.

Predictors were ranked by their mean bootstrap‐derived AUC. Reduced models were then constructed using: i) a chemokine‐only model with CXCL9 and CXCL10, ii) the top five features, iii) the top ten features, and iv) all 17 features. This comparison highlighted the predictive utility of EB‐measured CXCL9 and CXCL10 for point‐of‐care AR classification. By focusing on features with consistent discriminatory performance, the bootstrap selection strategy minimized non‐informative variables and improved model robustness.

## Conflict of Interest

The authors declare no conflict of interest.

## Author Contributions

R.G. and N.S. contributed equally to this work. R.G. performed conceptualization, data curation, methodology, formal analysis, validation, investigation, visualization, funding acquisition, and co‐wrote the original draft. N.S. contributed to methodology, investigation, validation, formal analysis, data curation, and co‐wrote part of original draft. A.K. was involved in methodology, formal analysis, and investigation. F.Y.C., M.J., A.A.K., and P.M. were involved in methodology and investigation. S.B. was involved in reviewing and editing, funding acquisition, resources, and conceptualization. R.M. and M.K.T. contributed to conceptualization, supervision, funding acquisition, reviewing and editing, and project administration.

## Supporting information



Supporting Information

## Data Availability

The data that support the findings of this study are available from the corresponding author upon reasonable request.

## References

[1] D. S. Keith , G. Vranic , A. Nishio‐Lucar , Transplant. Direct 2017, 3, 166. 10.1097/TXD.0000000000000654 PMC5464785 28620650

[2] M. Coemans , C. Süsal , B. Döhler , D. Anglicheau , M. Giral , O. Bestard , C. Legendre , M.‐P. Emonds , D. Kuypers , G. Molenberghs , Kidney Int. 2018, 94, 964. 30049474 10.1016/j.kint.2018.05.018

[3] H. Burton , L. Iyamu Perisanidou , R. Steenkamp , R. Evans , L. Mumford , K. M. Evans , F. J. Caskey , R. Hilton , Nephrol., Dial., Transplant. 2019, 34, 355. 29982787 10.1093/ndt/gfy168

[4] M. Pascual , T. Theruvath , T. Kawai , N. Tolkoff‐Rubin , A. B. Cosimi , N. Engl. J. Med. 2002, 346, 580. 11856798 10.1056/NEJMra011295

[5] A. Rana , A. Gruessner , V. G. Agopian , Z. Khalpey , I. B. Riaz , B. Kaplan , K. J. Halazun , R. W. Busuttil , R. W. Gruessner , JAMA Surg. 2015, 150, 252. 25629390 10.1001/jamasurg.2014.2038

[6] M. Gago , L. Cornell , W. Kremers , M. Stegall , F. Cosio , Am. J. Transplant. 2012, 12, 1199. 22221836 10.1111/j.1600-6143.2011.03911.x

[7] P. A. Clayton , S. P. McDonald , G. R. Russ , S. J. Chadban , J. Am. Soc. Nephrol. 2019, 30, 1697. 31308074 10.1681/ASN.2018111101 PMC6727270

[8] W. H. Lim , R. M. Turner , J. R. Chapman , M. K. Ma , A. C. Webster , J. C. Craig , G. Wong , Transplantation 2014, 97, 817. 24521777 10.1097/01.TP.0000442773.38510.32

[9] L. Henderson , B. Nankivell , J. Chapman , Am. J. Transplant. 2011, 11, 1570. 21797971 10.1111/j.1600-6143.2011.03677.x

[10] M. R. Laftavi , R. Stephan , B. Stefanick , R. Kohli , F. Dagher , M. Applegate , J. O'Keefe , D. Pierce , A. Rubino , H. Guzowski , Surgery 2005, 137, 364. 15746793 10.1016/j.surg.2004.10.013

[11] I. Rebollo‐Mesa , E. Nova‐Lamperti , P. Mobillo , M. Runglall , S. Christakoudi , S. Norris , N. Smallcombe , Y. Kamra , R. Hilton , S. Bhandari , Am. J. Transplant. 2016, 16, 3443. 27328267 10.1111/ajt.13932 PMC5132071

[12] J. Santos , L. S. Martins , World J. Nephrol. 2015, 4, 345.26167457

[13] P. N. Furness , N. Taub , Kidney Int. 2001, 60, 1998. 11703620 10.1046/j.1523-1755.2001.00030.x

[14] S. Abouchacra , A. Chaaban , R. Hakim , N. Gebran , H. El‐Jack , F. Rashid , Y. Boobes , A. Muhairi , Q. Hussain , I. Khan , Int. Urol. Nephrol. 2012, 44, 1871. 22639068 10.1007/s11255-012-0188-y

[15] P. Hirt‐Minkowski , P. Amico , J. Ho , A. Gao , J. Bestland , H. Hopfer , J. Steiger , M. Dickenmann , F. Burkhalter , D. Rush , Am. J. Transplant. 2012, 12, 1811. 22390571 10.1111/j.1600-6143.2012.03999.x

[16] M. Rabant , L. Amrouche , L. Morin , R. Bonifay , X. Lebreton , L. Aouni , A. Benon , V. Sauvaget , L. L. Vaillant , F. Aulagnon , Am. J. Transplant. 2016, 16, 1868. 26694099 10.1111/ajt.13677

[17] E. Mačionienė , M. Simanavičius , M. Vitkauskaitė , A. Vickienė , R. Staučė , A. Vinikovas , M. Miglinas , Ann. Transplant. 2024, 29, 944762. 10.12659/AOT.944762 PMC11490196 39402819

[18] I. Gandolfini , C. Harris , M. Abecassis , L. Anderson , O. Bestard , G. Comai , P. Cravedi , E. Cremaschi , J. A. Duty , S. Florman , Kidney Int. Rep. 2017, 2, 1186. 29270527 10.1016/j.ekir.2017.06.010 PMC5733675

[19] T. D. Blydt‐Hansen , A. Sharma , I. W. Gibson , C. Wiebe , A. P. Sharma , V. Langlois , C. W. Teoh , D. Rush , P. Nickerson , D. Wishart , Am. J. Transplant. 2021, 21, 1545. 33034126 10.1111/ajt.16336

[20] E. Van Loon , C. Tinel , H. de Loor , X. Bossuyt , J. Callemeyn , M. Coemans , K. De Vusser , V. Sauvaget , J. Olivre , P. Koshy , Am. J. Kidney Dis. 2024, 83, 467. 37777058 10.1053/j.ajkd.2023.07.022

[21] M. A. LeVatte , M. Lipfert , J. Zheng , D. S. Wishart , Anal. Biochem. 2019, 580, 1. 31153872 10.1016/j.ab.2019.05.015

[22] J. Wu , H. Liu , W. Chen , B. Ma , H. Ju , Nat. Rev. Bioeng. 2023, 1, 346. 37168735 10.1038/s44222-023-00032-w PMC9951169

[23] K. B. Patel , S. Luhar , D. N. Srivastava , Anal. Methods 2024, 16, 4971. 38973650 10.1039/d4ay00789a

[24] B. Jagannath , M. Pali , K. C. Lin , D. Sankhala , P. Naraghi , S. Muthukumar , S. Prasad , Adv. Mater. Technol. 2022, 7, 2101356.

[25] A. S. Tanak , S. Muthukumar , S. Krishnan , K. L. Schully , D. V. Clark , S. Prasad , Biosens. Bioelectron. 2021, 171, 112726. 33113386 10.1016/j.bios.2020.112726 PMC7569407

[26] K. L. Singampalli , C. Neal‐Harris , C. Yee , J. S. Lin , P. B. Lillehoj , Adv. Sens. Res. 2024, 3, 2400004. 39640072 10.1002/adsr.202400004 PMC11617009

[27] C. Jiang , G. Wang , R. Hein , N. Liu , X. Luo , J. J. Davis , Chem. Rev. 2020, 120, 3852. 32202761 10.1021/acs.chemrev.9b00739

[28] J. S. del Río , O. Y. Henry , P. Jolly , D. E. Ingber , Nat. Nanotechnol. 2019, 14, 1143. 31712665 10.1038/s41565-019-0566-z

[29] S. S. Timilsina , N. Durr , M. Yafia , H. Sallum , P. Jolly , D. E. Ingber , Adv. Healthcare Mater. 2022, 11, 2102244. 10.1002/adhm.202102244 34965031

[30] U. Zupančič , P. Jolly , P. Estrela , D. Moschou , D. E. Ingber , Adv. Funct. Mater. 2021, 31, 2010638.

[31] D. Najjar , J. Rainbow , S. Sharma Timilsina , P. Jolly , H. De Puig , M. Yafia , N. Durr , H. Sallum , G. Alter , J. Z. Li , Nat. Biomed. Eng. 2022, 6, 968. 35941191 10.1038/s41551-022-00919-w PMC9361916

[32] R. Gupta , N. Salaris , A. Kalkal , P. Mandal , S. Balabani , R. Motallebzadeh , M. K. Tiwari , IEEE Sens. Lett. 2024, 8, 1.

[33] R. Gupta , A. Kalkal , P. Mandal , D. Paital , D. Brealey , M. K. Tiwari , ACS Appl. Mater. Interfaces 2025, 17, 44112. 40704602 10.1021/acsami.5c06701 PMC12332829

[34] F. Tehrani , H. Teymourian , B. Wuerstle , J. Kavner , R. Patel , A. Furmidge , R. Aghavali , H. Hosseini‐Toudeshki , C. Brown , F. Zhang , Nat. Biomed. Eng. 2022, 6, 1214. 35534575 10.1038/s41551-022-00887-1

[35] J. Zhang , S. D. Petersen , T. Radivojevic , A. Ramirez , A. Pérez‐Manríquez , E. Abeliuk , B. J. Sánchez , Z. Costello , Y. Chen , M. J. Fero , Nat. Commun. 2020, 11, 4880. 32978375 10.1038/s41467-020-17910-1 PMC7519671

[36] E. F. Schisterman , D. Faraggi , B. Reiser , J. Hu , Stat. Med. 2008, 27, 297. 17624866 10.1002/sim.2993 PMC2749250

[37] K. Jangid , R. Gupta , R. P. Sahu , I. Zhitomirsky , I. K. Puri , J. Electroanal. Chem. 2022, 910, 116200.

[38] Y. Li , L. Luo , Y. Kong , S. George , Y. Li , X. Guo , X. Li , E. Yeatman , A. Davenport , Y. Li , Adv. Funct. Mater. 2024, 34, 2316865.

[39] Q. Wu , Q. Hou , P. Wang , C. Ding , S. Lv , ACS Appl. Mater. Interfaces 2023, 15, 44322. 37672622 10.1021/acsami.3c09737

[40] V. S. Vaidya , S. S. Waikar , M. A. Ferguson , F. B. Collings , K. Sunderland , C. Gioules , G. Bradwin , R. Matsouaka , R. A. Betensky , G. C. Curhan , Clin. Transl. Sci. 2008, 1, 200. 19212447 10.1111/j.1752-8062.2008.00053.x PMC2638059

[41] P. Fiorina , M. J. Ansari , M. Jurewicz , M. Barry , V. Ricchiuti , R. N. Smith , S. Shea , T. K. Means , H. Auchincloss Jr. , A. D. Luster , J. Am. Soc. Nephrol. 2006, 17, 716. 16481416 10.1681/ASN.2005090954

[42] J. A. Jackson , E. J. Kim , B. Begley , J. Cheeseman , T. Harden , S. D. Perez , S. Thomas , B. Warshaw , A. D. Kirk , Am. J. Transplant. 2011, 11, 2228. 21812928 10.1111/j.1600-6143.2011.03680.x PMC3184377

[43] S. Schaub , P. Nickerson , D. Rush , M. Mayr , C. Hess , M. Golian , W. Stefura , K. HayGlass , Am. J. Transplant. 2009, 9, 1347. 19459809 10.1111/j.1600-6143.2009.02645.x

[44] KRUK, Kidney disease: A UK public health emergency 2023.

[45] NHS, Annual report on kidney transplantation 2022.

[46] C. Tinel , A. Devresse , A. Vermorel , V. Sauvaget , D. Marx , V. Avettand‐Fenoel , L. Amrouche , M.‐O. Timsit , R. Snanoudj , S. Caillard , Am. J. Transplant. 2020, 20, 3462. 32342614 10.1111/ajt.15959

