# Supplementary material for: Electrochemical Biosensor for Rapid Detection of Acute Rejection in Kidney Transplants
Source: Adv Healthc Mater. 2025 Sep 4;15(1):e02831. doi: 10.1002/adhm.202502831 (PMC12790328; doi:10.1002/adhm.202502831)
Supplement: Supplementary file 1 — Supporting Information [file ADHM-15-0-s001.docx]

Supporting Information

Electrochemical Biosensor for Rapid Detection of Acute Rejection in Kidney Transplants

Rohit Gupta^1,2, #^, Nikolaos Salaris^1,2, #^, Ashish Kalkal^1,2^, Fernando Yuen Chang^3,4^, Maryam Javed^4^, Azhar Ali Khan^4^, Priya Mandal^1,2^, Stavroula Balabani^2,5^, Reza Motallebzadeh^3,4^, Manish K. Tiwari^1,2,6*^

^1^ Nanoengineered Systems Laboratory, UCL Mechanical Engineering, University College London, London WC1E 7JE, UK

^2^ UCL Hawkes Institute, University College London, London W1W 7TS, UK

^3^ Research Department of Surgical Biotechnology, Division of Surgery & Interventional Sciences, University College London, London, NW3 2PF, UK

^4^ Department of Nephrology & Renal Transplantation, Royal Free Hospital London, London, NW3 2QG

^5^ FluME, UCL Mechanical Engineering, University College London, WC1E 7JE London, UK

^6^ Manufacturing Futures Laboratory, University College London, London, E20 2AE, UK

^#^ The authors contributed equally

*Corresponding author details

Email: [m.tiwari@ucl.ac.uk](mailto:m.tiwari@ucl.ac.uk), Phone: +44 20 3108 1056 (Manish K. Tiwari)

| **Content** | **Page No.** |
| --- | --- |
| Appendix 1: Clinical dataset with 60 kidney transplanted patients | 3 |
| Appendix 2: Classification model workflow | 6 |
| Appendix 3: Albumin fouling tests on uncoated and coated electrodes | 7 |
| Appendix 4: Interference tests for CXCL9 and CXCL10 sensors | 8 |
| Appendix 5: Effect of model complexity on training and testing performance | 9 |

**Appendix 1: Clinical dataset with 60 kidney transplanted patients**

Clinical data are summarized using the following variables such as patient demographics, transplant characteristics, biopsy indications, primary pathological diagnoses, and clinical outcomes (Table S1). Statistical data are presented as either mean ± standard deviation (SD) or median with interquartile range (IQR), as appropriate. Clinical pathology data correspond to the day of biopsy, which was synchronized with urine collection performed prior to the biopsy procedure. The ABMR/TCMR classification is based on biopsy-confirmed acute rejection (AR) cases, following the Banff allograft pathology criteria. All remaining demographic and clinical data reflect values recorded on the day of kidney transplantation.

**Table S1.** Summary of the clinical dataset for diagnosing acute rejection with n = 60 kidney transplant recipients.

| **Variable Family** | **Variable Type** | **Description and sub-type** | **Value** |
| --- | --- | --- | --- |
| **Recipient characteristics** | Categorical | Male, n (%) | 36 (60%) |
|  | Continuous | Age (yr) at transplantation; mean ± SD | 54.1$\pm$ 13.7 |
|  | Categorical | Donor age (yr), mean ± SD | 50.5$\pm$ 13.6 |
|  | Categorical | White, n (%) | 24 (40%) |
|  |  | Asian, n (%) | 17 (28%) |
|  |  | Afro-Caribbean, n (%) | 16 (27%) |
|  |  | Other, n (%) | 2 (3%) |
| **Cause of ESRD** | Categorical | Autosomal dominant polycystic kidney disease (ADPKD) | 4 (6.7%) |
|  |  | Hypertension | 7 (11.7%) |
|  |  | Diabetes | 11 (18.3%) |
|  |  | Glomerulonephritis | 10 (16.7%) |
|  |  | Reflux Nephropathy | 3 (5.0%) |
|  |  | IgA Nephropathy | 6 (10.0%) |
|  |  | Drug-induced nephropathy | 1 (1.7%) |
|  |  | Other | 17 (28.3%) |
|  |  | Unknown | 1 (3.3%) |
| **Transplant Variables** | Categorical | DBD donor, n (%) | 21 (35%) |
|  | Categorical | DCD donor, n (%) | 27 (45%) |
|  | Categorical | Living donor, n (%) | 11 (18.3%) |
|  | Continuous | HLA mismatch level (1-4) *, median (IQR) | 3.0 (IQR: 2.0) |
|  | Continuous | Number of HLA mismatches **, median (IQR) | 8 (IQR: 3.0) |
| **Indication for biopsy and concomitant laboratory tests** | Continuous | Interval between transplant and biopsy (months), mean ± SD | 42.2$\pm$70.7 |
|  | Continuous | Rise in creatinine, n (%) | 59 (98.33%) |
|  | Continuous | Urinary creatinine (mmol/L), mean ± SD | 8.8 $\pm$ 13.8 |
|  | Continuous | Serum Creatinine (µmol/L), mean ± SD | 345$\pm$ 241 |
|  | Continuous | Urinary protein-to-creatinine ratio (mg/mmol), mean ± SD | 104.7$\pm$ 158.4 |
|  | Categorical | Presence of donor-specific antibodies (DSAs), n (%) | 45 (75%) |
|  | Categorical | Bacteriuria > 10^4 CFU/mL, n (%) | 17 (18.5%) |
| **Primary pathological diagnosis** | Categorical | Antibody-mediated rejection (ABMR), n (%) | 3 (5%) |
|  |  | T-cell mediated rejection (TCMR), n (%) | 12 (20%) |
|  |  | Mixed rejection, n (%) | 2 (3.3%) |
|  |  | Acute tubular necrosis (ATN), n (%) | 24 (40%) |
|  |  | Recurrent disease (e.g., IgA nephropathy, FSGS, Lupus), n (%) | 3 (5%) |
|  |  | BK virus-associated nephropathy, n (%) | 2 (3.3%) |
|  |  | Interstitial Fibrosis and Tubular Atrophy (IFTA), n (%) | 7 (11.7%) |
|  |  | Other, n (%) | 1 (1.7%) |
| **Clinical Outcomes** | Continuous | Serum creatinine (µmol/L) at last follow-up, mean ± SD | 212.4$\pm$ 83.8 |
|  | Categorical | Patient survival at last follow-up, n (%) | 54 (84.4%) |
|  | Categorical | Graft survival at last follow-up, n (%) | 54 (84.4%) |
|  | Continuous | Mean follow-up, years, ± SD | 5.2$\pm$ 5.8 |

*HLA mismatch level - level 1 was a 000 HLA-A, HLA-B, and HLA- DR mismatch; level 2 was a 0 HLA-DR plus 0/1 HLA-B mismatch; level 3 was a 0 HLA-DR plus 2 HLA-B mis- match or a 1 HLA-DR plus 0/1 HLA-B mismatch; and level 4 was a 2 HLA-DR or a 1 HLA-DR plus 2 HLA-B mismatch.

**Recipient sensitisation was defined as HLA antibody reaction frequency, which was calculated by comparison of unacceptable HLA specificities with HLA types of donors of identical ABO blood group in a pool of 10,000 donors on the UK transplant database.

**Appendix 2: Classification model workflow**

To assess the correlation and predictive power of the clinical data in relation to the biopsy results, a statistical analysis was performed, and a classification model was developed. The input features included the measurements from ELISA and EB sensing of the chemokines CXCL9 and CXCL10 and other relevant clinical data taken prior to the biopsy. The code developed has 3 main parts and uses open-source libraries. The code alongside the clinical data and an explanatory text file (README.txt) are provided. The flow chart is provided in Figure S1. The code and associated datasets can be found the following repository: <https://github.com/Nick-djsalas/Clinically-Relevant-Detection-of-Kidney-Transplant-Acute-Rejection-publication-cxcl-analysis/tree/main>


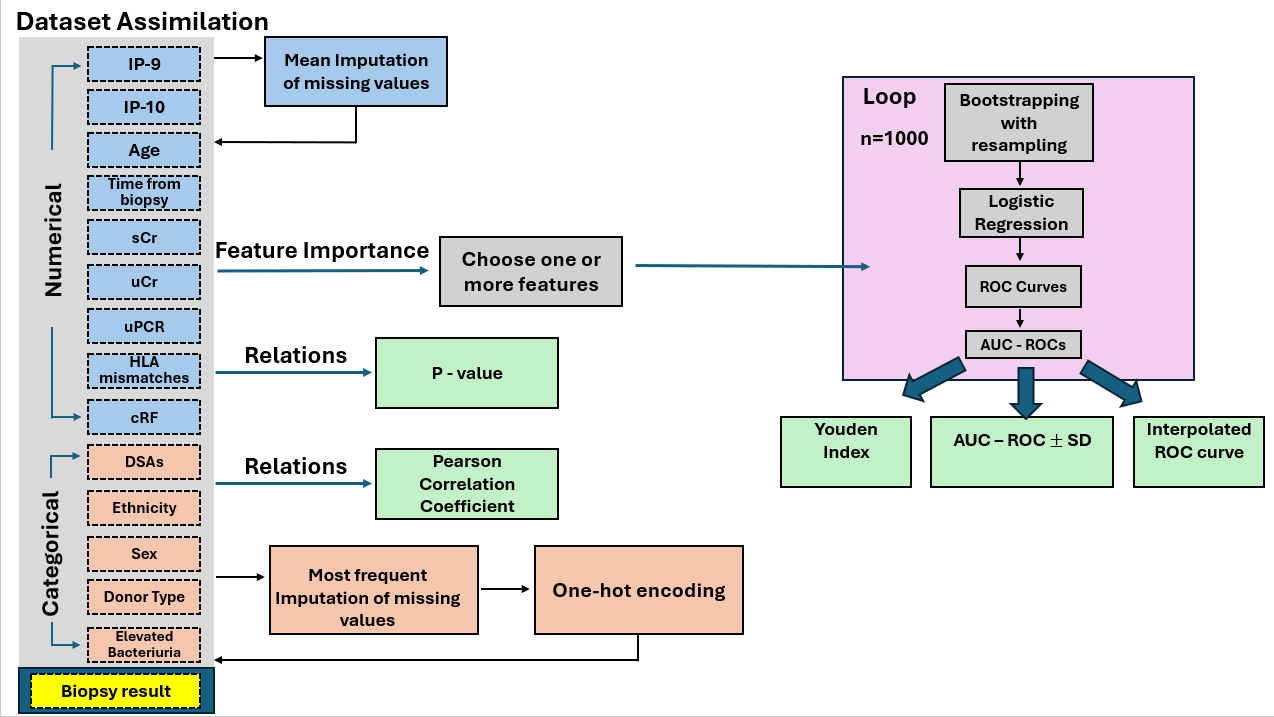


**Fig. S1**: Flow chart of the code implemented for to analyse the clinical data and develop the classification model for Acute Rejection prediction based on the features from the available clinical data.

**Appendix 3: Albumin fouling tests on uncoated and coated electrodes**


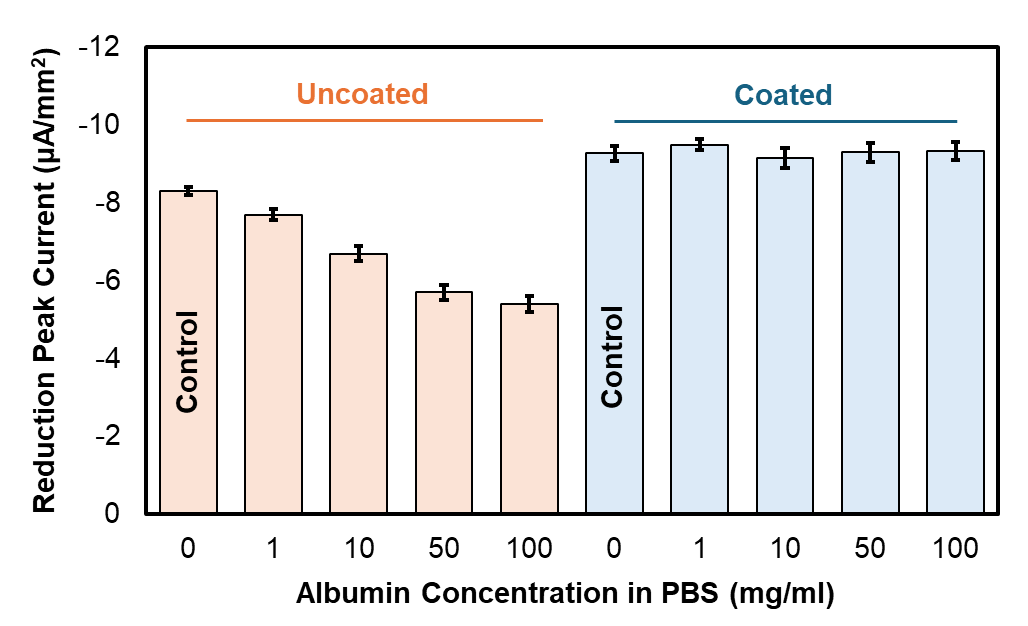


**Fig. S2.** Comparison of reduction peak currents for uncoated (bare screen-printed carbon) and hydrogel coated (MXene/BSA/GA) working electrodes exposed to different amount of albumin concentrations for a duration of 24 hr. For the coated electrodes, no statistically significant difference between different albumin concentrations were noticed. This shows that the coated electrodes resist non-specific protein adsorption and does not alter the Faradaic peak current for albumin concentrations as high as 100 mg/ml. Error bars represent ±1 SD for *n* = 3 independent electrodes. Significant difference was determined by unpaired two-tailed *t*-test assuming unequal variances.

**Appendix 4: Interference tests for CXCL9 and CXCL10 sensors**


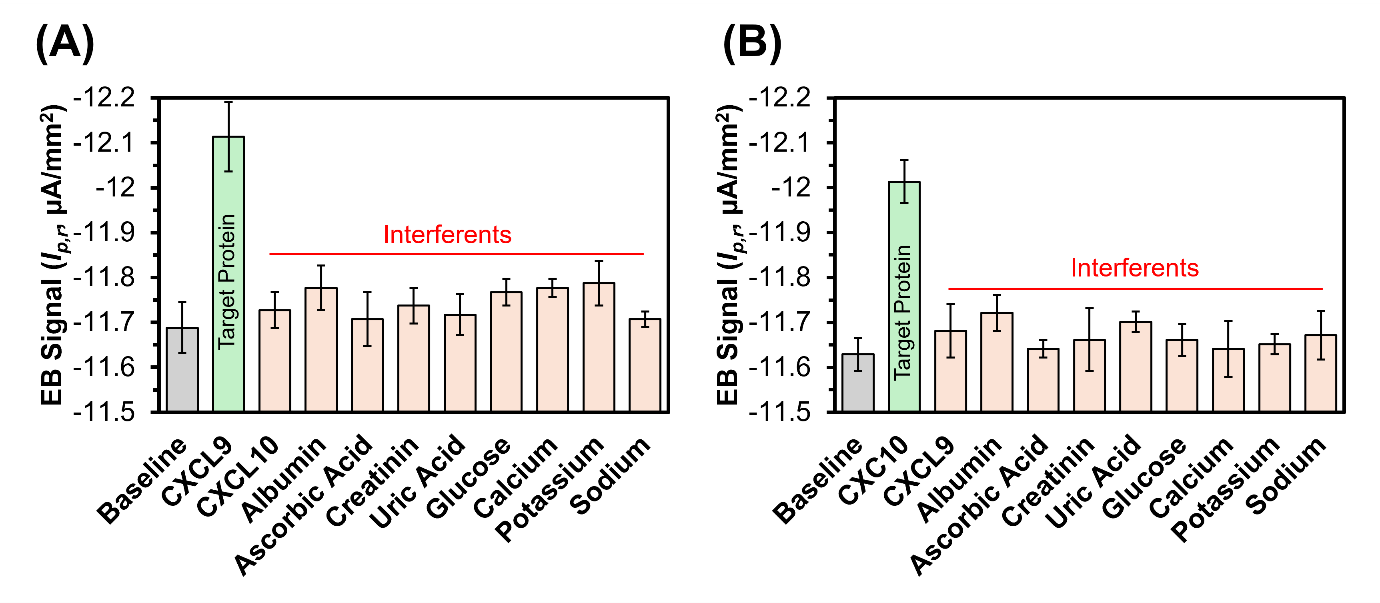


**Fig. S3.** Specificity tests for (A) CXCL9 and (B) CXCL10 sensors, comparing the signal acquired from the target chemokine and common interferents found in urine samples. The experiments were performed in triplicates and error bars represent ±1 SD of mean for *n* = 3 independent electrodes.

**Appendix 5: Effect of model complexity on training and testing performance**


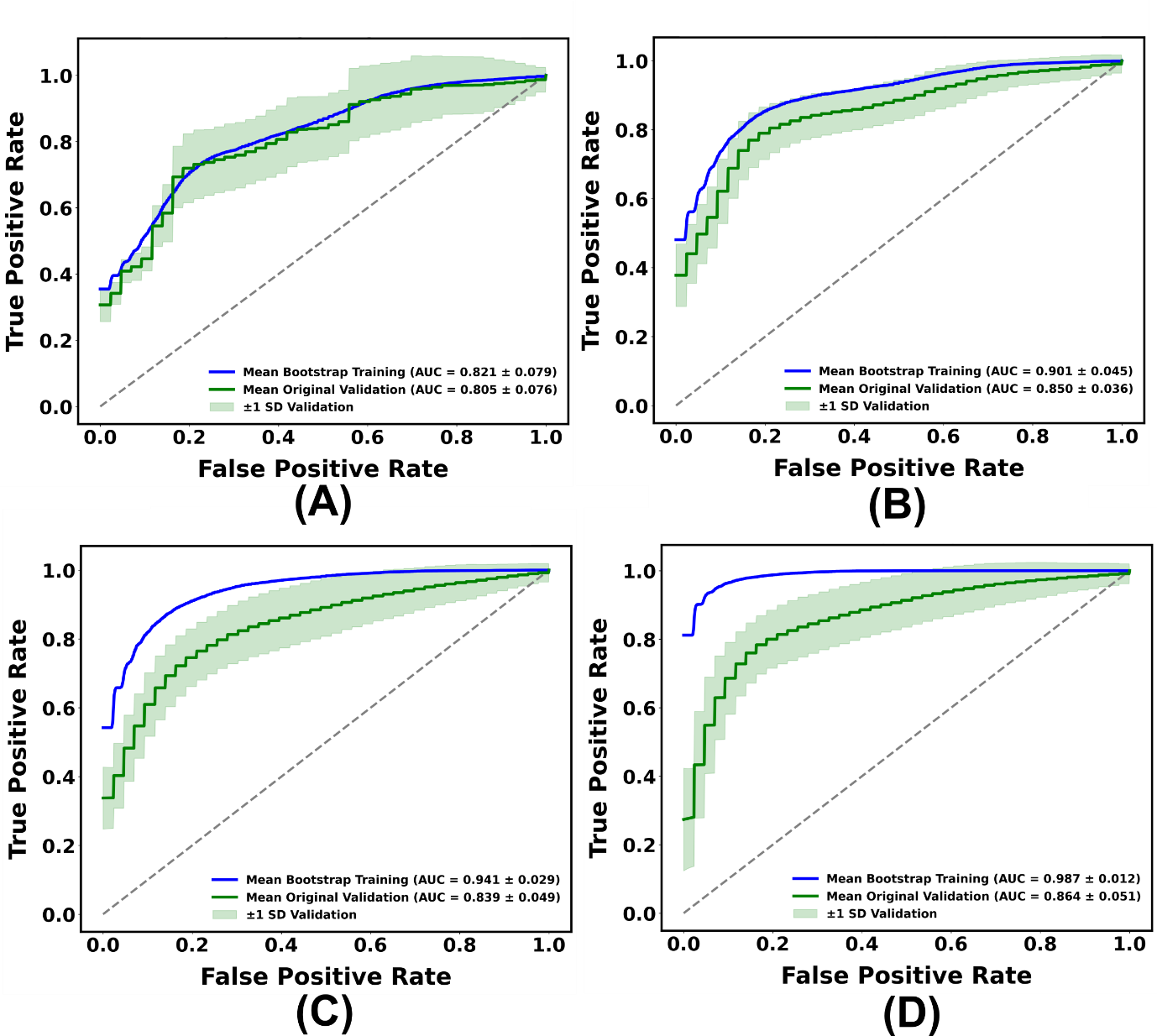


**Fig. S4:** ROC curves for logistic regression models of increasing complexity, evaluated using 1,000 repeats of 4-fold cross-validation. Each plot compares the mean performance on the training folds (blue line) with the mean performance on the testing (validation) folds (green line). The shaded green band represents ±1 SD of the testing performance. The panels display models built with: (A) the two chemokine predictors only, (B) the top 5 predictors, (C) the top 10 predictors, and (D) all 17 predictors (13 numerical and 4 categorical). The widening gap between the training and testing curves across the panels provides a clear visualization of increasing model overfitting as more features are included.
